# PARSEG: a computationally efficient approach for statistical validation of botanical seeds’ images

**DOI:** 10.1038/s41598-024-56228-6

**Published:** 2024-03-13

**Authors:** Luca Frigau, Claudio Conversano, Jaromír Antoch

**Affiliations:** 1 Department of Economics and Business Sciences, University of Cagliari, Viale S. Ignazio da Laconi 17, 09123 Cagliari, Italy; 2 Faculty of Mathematics and Physics, Charles University, Sokolovská 83, 186 75 Prague, Czech Republic; 3 Faculty of Informatics and Statistics, Department of Econometrics, Prague University of Economics and Business, Winston Churchill Square 1938/4, 130 67 Prague 3, Czech Republic

**Keywords:** Statistical image validation, Image segmentation, Background subtraction, Big data, Classification, CART, STAPLE, Bootstrap, Computational science, Statistics

## Abstract

Human recognition and automated image validation are the most widely used approaches to validate the output of binary segmentation methods but, as the number of pixels in an image easily exceeds several million, they become highly demanding from both practical and computational standpoint. We propose a method, called PARSEG, which stands for PArtitioning, Random Selection, Estimation, and Generalization; being the basic steps within this procedure. Suggested method enables us to perform statistical validation of binary images by selecting the minimum number of pixels from the original image to be used for validation without deteriorating the effectiveness of the validation procedure. It utilizes binary classifiers to accomplish image validation and selects the optimal sample of pixels according to a specific objective function. As a result, the computational complexity of the validation experiment is substantially reduced. The procedure’s effectiveness is illustrated by considering images composed of approximately 13 million pixels from the field of seed recognition. PARSEG provides roughly the same precision of the validation process when extended to the entire image, but it utilizes only about 4% of the original number of pixels, thus reducing, by about 90%, the computing time required to validate a binary segmented image.

## Introduction

Images of biological objects, botanic seeds in our case, contain enormous amounts of information, which can be extracted and used as input for the subsequent analyses. To extract a piece of information from an image, it is necessary to preprocess it using the tools of image analysis^1^. The preprocessing consists of several phases, among which image segmentation is one of the most important as it involves splitting an image into the parts that are strongly associated with real objects of interest^2,3^. Process of image segmentation constitutes a never ending challenge. Unfortunately, any segmentation methods suffers some drawbacks, typically connected to limited accuracy, excessive complexity and exaggerated time and space requirements. One possible solution, in many situations considered as a golden standard, is the use of human raters. However, it is usually quite costly because it is not easy to train them and, what is worse, to maintain their high uniform level during the long time horizon. Moreover, we can meet both intra- and extra- variability due to their fatigue, especially when dealing with extensive data sets.

A specific case is binary image segmentation, where the image is divided into two parts, called foreground and background, which correspond to the parts we are and are not interested in. Despite the great progress in this field, binary segmentation is still one of the most challenging tasks in image processing, image understanding, artificial intelligence^4^ and big data^5–7^.

As segmentation algorithms may lack accuracy and precision, as well as ground truth is frequently missing, assessing their performance is a difficult task. This assessment is of key importance, especially when its output is later analyzed statistically, because the results of statistical analyses are, to a considerable degree, influenced by the quality of the input data. The method, or set of methods, to be used to compare segmentation approaches has not yet been clearly defined; several methods are used in practice. The most common method to assess the quality of image segmentation is the interactive drawing of the image by experts. However, it cannot be considered reliable because, besides intra- and inter-expert variability, it is labour-intensive, subjective, and often suffers from inconsistencies and errors. Alternatively, computer-aided automatic methods can serve this purpose: although they should remove the variability of assessments, they are not always able to provide reliable results. The common problem in characterizing both human experts and automatic methods is that the true segmentation of the image is unknown, particularly in the case of medical images, in which the true segmented image might vary from case to case since the same pathology can appear from different forms or shapes.

One feasible alternative to human recognition is statistical validation of the performance of image segmentation algorithms. In statistics, validation is the task of confirming that the outputs of a statistical model are acceptable with respect to the real data-generating process. In image analysis, statistical validation is a process aimed at confirming that the output of an image segmentation method is accurate. If statistical validation provides reliable results, it is very likely that the considered image segmentation method is, with maximum reliability, able to reproduce the main features of the analyzed image. To account for the above-mentioned drawbacks derived from human recognition, an automatic and effective procedure has been proposed in^8^. It aims at the statistical validation of the outcomes provided by the binary segmentation of images based on statistical classification algorithms. Such a validation procedure is typically performed on very large data sets, inasmuch as the number of pixels in an image easily exceeds millions. The computational complexity of the validation experiment of segmented images is thus very high. To reduce this complexity, we present here a method called PARSEG, which comprises the following data-processing steps: PArtitioning, Random Selection, Estimation, and Generalization. PARSEG enables us to perform statistical validation of binary images by selecting the minimum number of pixels from the original image to be used for validation without deteriorating the effectiveness of the validation procedure. PARSEG overcomes the computational complexity of statistical image validation. The basic motivation supporting the use of PARSEG is derived from our empirical experiments: the results of statistical validation of binary segmentation methods, obtained by training a classifier on all pixels of the analyzed image, are consistent with those obtained using much smaller randomly selected samples of pixels of a specific size. This equivalence leads to a considerable decrease in the computational complexity of validation for binary segmentation of images comprising millions of pixels when using PARSEG.

 The selection of the optimal sample of pixels is derived from a properly selected objective function, which must be minimized to reduce the computational complexity of the validation procedure (see Section “Objective function” for details). Operationally, PARSEG is based on a sampling scheme that allows us to select a reduced number of pixels and, at the same time, preserves a sufficient scope of information needed for the subsequent image validation (see Section “Data partitioning and random subset selection” for details). Firstly, the entire image is partitioned into subsets of pixels of approximately equal size. Secondly, the minimum sample size of pixels to be extracted at random from a single subset is identified. This optimal reduced size should, as much as coherence, preserve the same amount of information as the original (complete) data used in the image validation process. The optimal size is selected via the study of the (functional) relationship comprising variations of possible sample sizes and the predictive performance of an appropriate classifier, selected by the user (see Section “Consistency measure” for details). Next, during the generalization step, validation based on statistical classifiers is performed independently on the remaining subsets using solely a sample of pixels with the previously identified optimal size. Finally, the results obtained from all subsets are combined to assess the validation’s effect on the entire image (see Section “Selection of the optimal sample size” for details).

The effectiveness of PARSEG is demonstrated through examples from the biology of plants, namely, the classification of seeds from the genome bank. Recall that, in the two most recent decades, many specialists in the botanical taxonomy field testified to the growing importance of the biometric features obtained by computer vision techniques employed in the characterization and identification of plant species^9–11^, varieties^12,13^, or identification of ancient plants^14,15^. Within this framework, the main initial point of interest is to correctly separate the pixels into a foreground and a background. Since there is no single method that can be recommended as the preferable one for all types of images, it is necessary to compare different binary segmentation procedures, enabling one to select “the most suitable one”^16^. This uncertainty is considered in our experiments as the different segmentation methods are ranked w.r.t. their performance from the most to the least accurate (see Section “*Giallo Bosa* example”).

The paper is organized as follows. Binary thresholding and its statistical validation are concisely discussed in Section “Binary thresholding and assessing its quality via statistical validation”. PARSEG, its main features, objective function, and key procedures are explained in Section “PARSEG”. Section “Comparison between PARSEG and STAPLE” illustrates a comparison between PARSEG and the *Simultaneous Truth and Performance Level Estimation* algorithm (STAPLE), a similar approach presented in literature. Section “Validating binary segmented seed images” illustrates the results of our approach applied to the analysis of real data (binary-segmented seed images), together with the discussion of the corresponding pros and cons. Finally, Section “Concluding remarks” provides the main conclusions of the paper and Section 7 plans for future work.

## Binary thresholding and assessing its quality via statistical validation

In mathematics, an image can be modeled by a continuous function of two variables *f*(*x*, *y*), where (*x*, *y*) are the coordinates in the plane (usually pixel indices). If the image is in grayscale, then $f(x,y) \rightarrow [0,1]$ is a scalar function, and it has three or four dimensions if the image is in a color mode. Depending on the combinations of the primary colors used, it is possible to decide between different color spaces, among which the most common are RGB and CMYK. In this paper, we deal with RGB images. Consequently, $f:(x,y) \rightarrow (R_{x,y}, G_{x,y}, B_{x,y})$, where $(R_{x,y}, G_{x,y},B_{x,y}) \in [0,1]^3$, and $R_{x,y}, G_{x,y}$ and $B_{x,y}$ represent intensities of the red, green and blue color channels for a given pixel (*x*, *y*), respectively.

The statistical validation method we propose here can be applied to any image segmentation method. However, for simplicity of our exposition, we focus on one of the most commonly used: *grey level thresholding* (see^17^ for an adaptive approach).

Recall that thresholding can be interpreted as a transformation of an image *f* into a binary image *o*, where

1 $$\begin{aligned} o(x,y)={\left\{ \begin{array}{ll} 0, &{} \quad f(x,y) < T(x,y), \\ 1, &{} \quad f(x,y) \ge T(x,y), \end{array}\right. } \end{aligned}$$

*T*(*x*, *y*) is the threshold value for pixel (*x*, *y*), $o(x,y)=1$ stands for the foreground pixel, and $o(x,y)=0$ for the background one^1^. The main critical task of this method is the selection of a correct threshold, which is essential for a successful segmentation and subsequent analysis. To this purpose, it is possible to use global or local information and, consequently, to decide between global and local thresholding. *Global thresholding* consists of finding a single threshold *T* for the entire image, i.e., $T(x,y)=T$ $\forall x,y$; whereas, *local thresholding* utilizes a threshold value *T*(*x*, *y*) for each pixel separately based on the information about its neighbors.

Our approach to the validation of the output produced by any binary image segmentation method is based on statistical modeling; hence the term *statistical validation* is used ^18^. Some approaches to validation (like^19^) are aimed at defining membership functions based on image descriptors in an alternative to the classical histogram-based image descriptors. Likewise, statistical validation is carried out using a classification experiment whose results are evaluated through a coherence index enabling us to check for the quality of the binary segmentation outcome^8^.

The main features of a statistical validation experiment in the case of grey-level thresholding segmentation (these features characterize any image segmentation method) are:

1. The labels assigned by a specific binary image segmentation method, either foreground or background, are used as binary response variables for a statistical classifier. This means that pixels are re-classified into one of the two categories on the basis of the corresponding RGB intensities to derive the “validated labels”.
2. As for the assessment of the classifier’s performance, it is possible to use a metric that compares pixel-wise observed labels with the predicted ones. This metric might be, in a specific case, that of accuracy, sensitivity, specificity, positive predictive value, or Area Under the ROC Curve (see^20,21^ for a discussion).
3. The selected metric is then used to evaluate the quality of the validation experiment by ranking the alternative image segmentation algorithms. The higher the accuracy level of the classifier, or the higher the correspondence between labels obtained from the image segmentation algorithm and label predicted by the classifier, the higher the image segmentation algorithm is ranked. If this is the case, the validation experiment produces satisfactory results and the image segmentation method is considered reliable for the assignment of the “validated” label (background or foreground) to each pixel.

## PARSEG

We provide a step-by-step description of PARSEG illustrating every single step and the main issues characterizing the resulting validation experiment.

### Objective function

We denote by $r_s$ a sample of pixels of size *s* randomly drawn from the entire image, and by $\mathscr {S}= \big \{s_1,s_2,\ldots ,s_{tot}\big \}$ a pre-specified set of sample sizes ($s_i\in \mathbb {N}$ such that $s_i<s_j$ if $i<j$) with $s_{tot}$ indicating the total number of pixels in a given image. Let $\psi _s$ be the index measuring the difference in terms of consistency (i.e., numerical coherence, to be explained in detail in Section “Consistency measure﻿”) between the validation results obtained on $r_s$ and on $r_{tot}$; $\psi _s$ decreases when *s* increases, and

$$\begin{aligned} h:s\rightarrow \psi _s,\qquad \forall s\in \mathscr {S}, \end{aligned}$$

is the function describing the relationship between *s* and $\psi _s$; from an empirical study based on our data it emerged that *h* tends to be monotonically decreasing since $\psi _s$ monotonically decreases on average when *s* increases.

The search of the “optimal” minimum sample size, say $s^*$, is aimed at compensating for the relative increase in complexity observed when moving from $s_i$ to $s_{i+1}$ with the relative decrease in the difference $\big |\,\psi _{s_{i+1}}-\psi _{s_i}\,\big |$. Thus, $s^*$ is defined as

2 $$\begin{aligned} s^* = \arg \min _{s \in \mathscr {S}} \big |\, h^{\prime }(s)+1 \,\big |, \end{aligned}$$

where $h^{\prime }$ denotes a derivative of *h*. In practice, given a set of samples $\big \{r_{s_i}\big \}_i$, the optimal point $\big (s^*,\psi _{s^*}\big )$ corresponds to that point for which $h^{\prime } \approx -1$.

### Data partitioning and random subset selection

To combine the original RGB image *f* with the corresponding binary image *o*:

1. the *N* pixels of *f* are organized into a set $\textbf{x} = \{ \textbf{x}_1, \ldots , \textbf{x}_N\}$: each $\textbf{x}_i$ contains the three values representing RGB color channel intensities of the pixel *i*;
2. identical pixels of *o* are arranged in $\textbf{y} = \big (y_{1}, \ldots , y_{N}\big )$;
3. $\textbf{x}$ and $\textbf{y}$ are joined to create a new set $\mathscr {D} = \big \{\big (\textbf{x}_1,y_{1}\big ), \ldots , (\textbf{x}_N,y_{N})\big \}$.

$\mathscr {D}$ is a collection of *N* pairs containing the information about both the original pixels of *f* (the input) and *o* (the output). Next, $\mathscr {D}$ is partitioned into *M* mutually disjoint subsets $\mathscr {D}_1,\ldots ,\mathscr {D}_M$ ($\bigcup _{j=1}^M\,\mathscr {D}_j = \mathscr {D}$) using a random sample stratified by $\textbf{y}$. Consequently, the *M* subsets (of cardinality $n \approx N/M$) are characterized by a similar distribution of the categories of $\textbf{y}$ and an unknown function that maps $\textbf{x}$ to $\textbf{y}$.

### Validation

To validate a binary segmentation method, one subset $\mathscr {D}_j \in \{\mathscr {D}_1,\ldots ,\mathscr {D}_M\}$ is randomly selected and next validated. To reduce computational complexity, a subsample $r_{js}$ of size $s,\ s\in \mathscr {S}$, is drawn from $\mathscr {D}_j$, and the pixels in $r_{js}$ are randomly partitioned into a learning set $t_{js}$ of cardinality $|t_{js}|$ and a validation set $|v_{js}|$ of cardinality $v_{js}$, such that $r_{js} = t_{js} \cup v_{js}$, $t_{js} \cap v_{js} = \emptyset$, and $\pi =|t_{js}|/|v_{js}|$ is the ratio between the two cardinalities.

Next, the $\textbf{y}_{js}^{v}$ pixels of the validation set $v_{js}$ are validated by computing predicted outcome $\widehat{\textbf{y}}_{js}^{v} = \mathscr {C}(t_{js}, v_{js} | \kappa )$ using an appropriate classifier $\kappa$. The function $\mathscr {C}$ utilizes the observations of the learning set $t_{js}$ to train $\kappa$ and estimates $\widehat{\textbf{y}}_{js}^{v}$ for the observations in the validation set $v_{js}$. In our experiments, although it is possible to consider any alternative metric, *sensitivity* (sometimes also called the true positive rate, recall, or probability of detection) is used as the reference classifier performance metric since it has been empirically confirmed as a reliable metric in statistical validation experiments. It is defined as

3 $$\begin{aligned} \phi _{js}=\frac{{(\widehat{\textbf{y}}_{js}^{v})^{\top } \widehat{\textbf{y}}_{js}^{v}}}{{(\textbf{y}_{js}^{v})^\top \textbf{y}_{js}^{v}}}; \end{aligned}$$

$\phi _{js}$ is computed for each possible sample size $s \in \mathscr {S}$ of the randomly selected subset $\mathscr {D}_j$. Moreover, to take into account model instability, the influence of outliers, and possible variable selection bias, the function $\mathscr {C}(\cdot )$ in PARSEG is estimated *B* times for each size $s \in \mathscr {S}$, each time with a different random partition of $r_{js{(b)}}$ into $t_{js{(b)}}$ and $v_{js{(b)}}$. In view of that, for a sample $r_{js}$ drawn from the partition $\mathscr {D}_j$, the performance of $\mathscr {C}(\cdot )$ is evaluated in terms of the average sensitivity

4 $$\begin{aligned} \bar{\phi }_{js} = B^{-1}\sum \nolimits _{b=1}^B {\phi }_{js{(b)}}. \end{aligned}$$

### Consistency measure

The basic idea supporting PARSEG is the selection of the “optimal” size $s^*$ as the smallest size $s \in \mathscr {S}$ that ensures for $\bar{\phi }_{js}$ to be consistent with $\bar{\phi }_{jn}$ (where *n* is the total number of elements of $\mathscr {D}_j$). To measure the difference in terms of consistency between $\bar{\phi }_{js}$ and $\bar{\phi }_{jn}$, we consider the index

5 $$\begin{aligned} \psi _{js} = |\bar{\phi }_{js} - \bar{\phi }_{jn} |\cdot |\sigma _{\phi _{js}} - \sigma _{\phi _{jn}} |, \end{aligned}$$

where

6 $$\begin{aligned} \begin{aligned} \sigma _{\phi _{js}}&= \sqrt{\tfrac{1}{B-1}\sum \nolimits _{j=1}^B \left( \phi _{js{(b)}} - \bar{\phi }_{js} \right) ^2}, \\ \sigma _{\phi _{jn}}&= \sqrt{\tfrac{1}{B-1}\sum \nolimits _{j=1}^B \left( \phi _{jn{(b)}} - \bar{\phi }_{jn} \right) ^2} \end{aligned} \end{aligned}$$

represent, respectively, the standard deviations of the values $\phi _{js{(b)}}$ and $\phi _{jn{(b)}}$, $b=1,\ldots ,B$. Eq. (5) is made up of two terms: $|\bar{\phi }_{js} - \bar{\phi }_{jn} |$ evaluates how much the sensitivity obtained for $r_{js}$ differs from that obtained for $\mathscr {D}_j$, which is the highest one. The second term, $|\sigma _{\phi _{js}} - \sigma _{\phi _{jn}} |$, weighs the first term with respect to the higher estimation uncertainty derived from the use of a sub-sample $r_{js}$ in place of the entire set of observations $\mathscr {D}_j$. For any $\bar{\phi }_{js}>0.5$, an increase in the sample size *s* is likely to cause the classifier $\mathscr {C}$ to be more accurate; it means that it will decrease the value of $\psi _{js}$.

### Selection of the optimal sample size

The search for $s^*_j$ through objective function (Eq. 2) should be carried out after estimating $\widehat{\psi }_{js}$ for each reduced sample $r_{js}$, $s\in \mathscr {S}$. To further reduce computational complexity, we consider the efficient approach summarized in Algorithm 1. It requires two user-defined input parameters, *l* and $\gamma$. The first is the minimum number of sample sizes in which to search for the optimal one in the first iteration, that is, $(l+1)$. In iteration *i*, the optimal sample size $s^*_j$ is searched for in a subset of possible sample sizes $\xi _i=\{s_1,s_2,\ldots ,s_l,s_{(l+i)},n\}$ composed of the first $(l + i)$ elements of $\mathscr {S}$ plus the maximum size (*n*); it stops when the same (optimal) sample size is found for $\gamma$ consecutive iterations.

Next, the index $\psi _{js}$ is computed for each sample size belonging to $\xi _i$ and the function *h* describing the relationship between the standardized values of the sample sizes, i.e., $\delta (\xi _i)$, and the standardized values of the $\psi _{js}$ index, i.e., $\delta (\Psi _{i})$, is fitted. The optimal sample size is found by applying the objective function (Eq. 2). If the number of times $\alpha$ in which the last optimal sample size is equal to the optimal sample sizes found in the previous $\gamma$ iterations, the algorithm stops, otherwise it keeps running.

**Algorithm 1:**
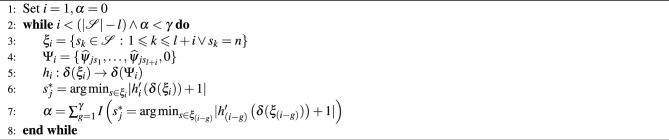
Selection of the optimal sample size

Once $s^*_j$ is defined for a given subset $\mathscr {D}_j$, it can be used as the reference sample size for the other $M-1$ subsets because, due to the stratified sampling scheme described in Section “Data partitioning and random subset selection﻿”, the response classes and the RGB intensities have the same distribution as that prevailing in the entire image. In particular, the same distribution of the response classes in the *M* subsets is guaranteed because the subsets are created by randomly partitioning all pixels with the constraint of having the same proportions of foreground pixels (and consequently also of background ones) as in the entire image. The same distribution of RGB intensities in the *M* subsets, instead, is deduced from the randomness that regulates the process of assigning the pixels to each subset inasmuch we assume that the pattern describing the relationship between the response classes and the RGB intensities is identical everywhere in the image. Consequently, $M-1$ samples $r_{ms^*_j}$ ($m=1,\ldots ,M,\ m\ne j$) are drawn from the subsets $\mathscr {D}_1,\ldots ,\mathscr {D}_M$ and the metric $\phi _{js}$ (Eq. 3) is computed in each subset $\mathscr {D}_j$. Next, $\phi _{js}$ is extended for the entire image $\mathscr {D}$ by averaging its values over the *M* subsets

7 $$\begin{aligned} \bar{\phi }_{\mathscr {D}} = M^{-1} \sum \nolimits _{m=1}^M \bar{\phi }_{ms^*_j}. \end{aligned}$$

In the next Section, we apply PARSEG to the images of botanic seeds. PARSEG provides roughly the same precision of the validation process extended to the entire image composed of *N* pixels but, importantly, it consistently reduces the computational complexity from *O*(*N*) to $O(M\cdot {s}_j^{*})$ with $M\cdot {s}_j^{*}\ll N$.

It is important to note that the segmentation method to be evaluated has to be carried out at the beginning of the process only. At each step, PARSEG uses solely pixels from the set $\mathscr {D}$, which contains the pixel intensities and their corresponding binary outputs defined by the underlying segmentation method.

## Comparison between PARSEG and STAPLE

Despite of PARSEG is more concentrated on the computational part of the statistical validation of images with the aim of selecting the best segmentation between those considered, its final goal is to provide a segmentation to be used as the best one. Consequently, in this Section we provide a comparison between the output obtained by PARSEG and that obtained by another method accepted in literature^22–25^. As evident from the citation report in both Web of Science and Google Scholar, the STAPLE algorithm^22^ is a widely accepted method for the statistical validation of image segmentation due to its sound theory and ease of use. STAPLE quantifies the performance of image segmentation raters (human or algorithmic) without knowing the true foreground, and is considered particularly useful in cases in which it is difficult to obtain or estimate a known true segmentation. It considers a set of segmentation outputs of an image, and estimates, for each of them, the probability of being the true segmentation. The latter is estimated to create an optimal combination of the segmentation options by weighing them according to their estimated performance level and by incorporating a prior model that considers the spatial distribution of the segmented structures and the spatial homogeneity constraints.

Both STAPLE and PARSEG pursue the goal of finding the best segmentation without knowing the true one: the former by generating a new segmentation from the optimal combination of the original ones, the latter by finding the best segmentation among those available. Furthermore, both methods define a relative performance measure of the original segmentation options according to their proximity to the best one. But, they operate in a different manner: STAPLE identifies the best segmentation by comparing the original segmentation options and the prior information available (if any); PARSEG searches for the patterns that link the original images (i.e., the color channel intensities) to the segmentation options, without referring its analysis to any comparison. Consequently, STAPLE performance could suffer if the segmentation set contains many wrong segmentation outputs and few correct ones. Instead, since PARSEG is not based on a comparison among the segmentation outputs, its performance is not influenced by the presence of a wrong segmentation. However, if the initial segmentations are wrong, neither PARSEG nor STAPLE can improve as is well known not only in statistics, but also in computing and other fields. Incorrect or poor-quality input will produce faulty output (garbage-in, garbage-out).

Concerning the computational requirements, we have assessed that both are linear in the number of segmentation outputs to be evaluated. Moreover, PARSEG is linear in the optimal size times the number of partitions, that is, $M\cdot s_j^*$, whereas STAPLE is linear in the number of pixels *N*. Being $M\cdot s^*_j \ll N$, PARSEG allows for important computational savings.

## Validating binary segmented seed images

We present detailed results obtained by applying PARSEG on the images of the seeds of species *Giallo Bosa* and summarize more concisely the results obtained for a set of sixteen different images of different seed species, including *Giallo Bosa*. We used data collected in previous studies^10,26^. The seeds were gathered by the authors of these studies^10,26^ from 16 traditional Sardinian cultivars from the CNR-ISPA field catalogue (Nuraxinieddu, Sardinia, Italy) (Table 1) and stored at the *Banca del Germoplasma Sardo* (BG-SAR) of the University of Cagliari. The mature fruits were collected randomly in order to obtain representative samples while reducing the impact of intra-specific variations in seed shapes and sizes caused by fruit position on the plant and seed position within the fruit.

**Table 1:** General information about seeds gathering.

	Species	Sampling location	Number
1	*Cariadoggia*	*Alghero*	80
2	*Cariasina*	*Medio Campidano*	39
3	*Coru*	*Laconi*	55
4	*Coru e Columbu*	*Laconi*	80
5	*Croccorighedda*	*Laconi*	30
6	*Fara*	*Bonarcado*	30
7	*Giallo Bosa*	*Bosa*	30
8	*Laconi A*	*Laconi*	87
9	*Melone*	*Gonnosfanadiga*	77
10	*Mirabolano Giallo*	*	90
11	*Mirabolano Rosso*	*	75
12	*Nero Sardo*	*Bosa*	99
13	*San Giovanni*	*Oristano*	39
14	*Sanguigna I Bosa*	*Bosa*	85
15	*Shiro*	*	94
16	*Sighera*	*Gonnosfanadiga*	88

*Stands for commercial species.

This data was collected with the goal to develop a suitable methodology allowing us to discriminate between seeds as well as possible. This is an important task from a quality control standpoint: one of the most important ways to enhance food quality is to guarantee the origin of different food products by traceability, which is able to identify responsibilities, optimize the supply chain, and ensure consumer food safety. Simply relying on documentation does not guarantee the truthfulness of the product’s origin. Thus, it is essential to develop instruments that give us a higher degree of reliability. Since seeds are among the most important raw materials in the agri-food market, discrimination among them is crucial to understand their origins.

### *Giallo Bosa* example

**Figure 1:**
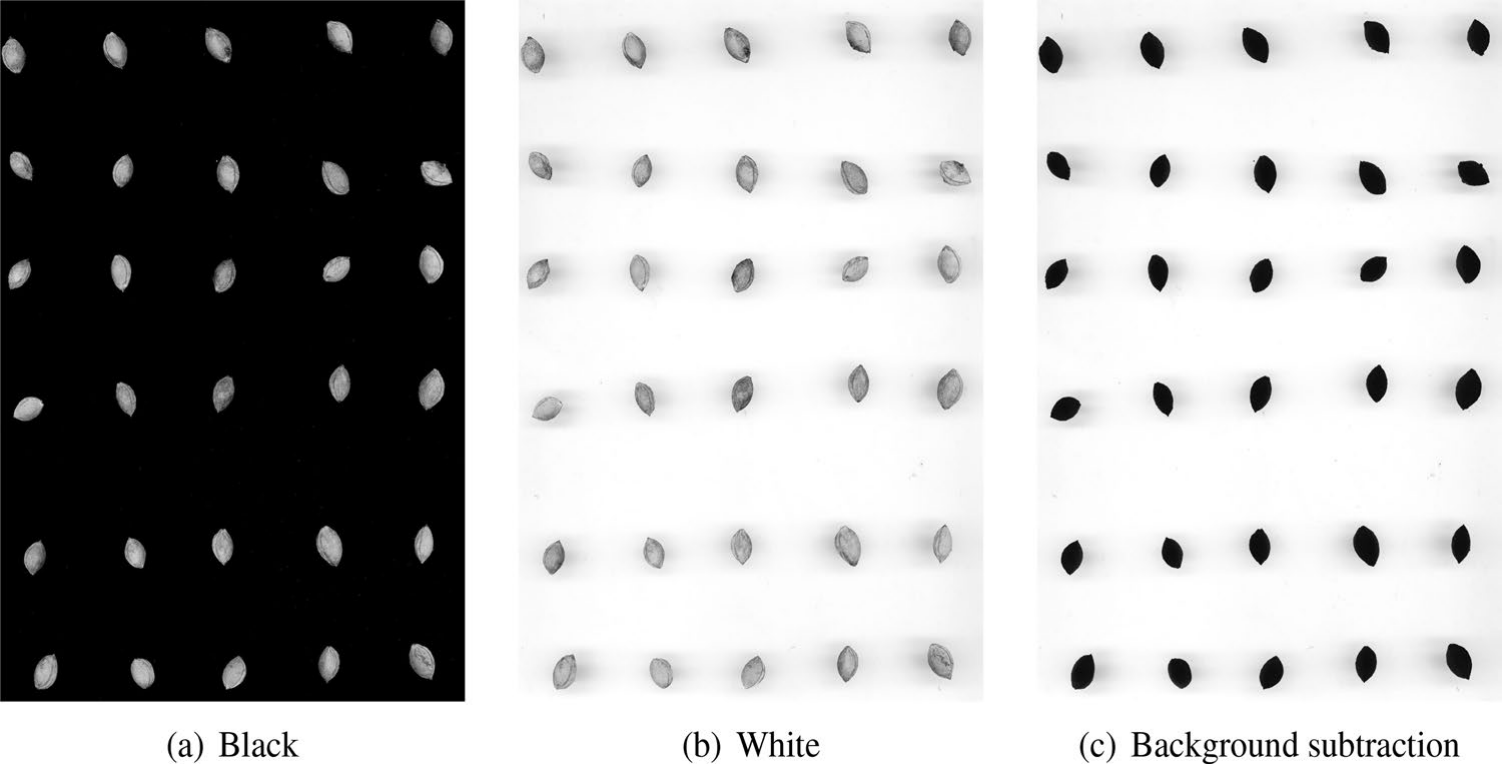
Image of the *Giallo Bosa* seeds captured using : (**a**) black background; (**b**) white background; (**c**) image resulting from background subtraction described in^8^.

The RGB images of the seeds *Giallo Bosa* are captured twice using a black background and a white background, in both cases without changing the position of the seeds, with a resolution of $4\,251 \times 2\,994$ ($N= 12\,727\,494$) pixels. Next, the background subtraction approach is applied, resulting in a new image, serving as an input for binary segmentation algorithms. Recall that background subtraction is a method widely used for detecting moving objects from a video, which has been adapted and modified for image segmentation in^8^. It combines local and global thresholding techniques to take advantage of the computational efficiency of the former and the accuracy of the latter, provides good results in segmentation, and allows for automating the process when the foreground color of images is not constant. Moreover, it is able to speed up computations quite significantly. All the algorithms listed in Table 2 are applied to separate the foreground, i.e., the seeds, from the background. Since all these algorithms require one-dimensional input, the input image provided by the background subtraction approach is first converted from the RGB to the grey scale (see Fig. 1). Finally, the morphological operators erosion and dilation (described in^27^) are used to enhance the binary segmentation output’s quality.

**Table 2:** The most widespread and the most frequently used binary segmentation algorithms.

Segmentation algorithm	References	Label
Adaptive document image binarization	^28^	Sauvola
Alternative implementation of Huang’s method	^29^	Huang2
Huang’s fuzzy thresholding method	^30^	Huang
Intermodes	^31^	Intermodes
Mean of gray levels	^32^	Mean
Means of image thresholding	^33^	Shanbhag
Minimum	^31^	Minimum
Otsu’s threshold	^34^	Otsu
Renyi’s entropy threshold	^35^	RenyiEntropy
Similarity-invariant pattern recognition	^36^	Percentile
Triangle method	^37^	Triangle
Tsai’s method	^38^	Moments

To validate the output of the different binary segmentation algorithms with PARSEG, the input parameters are set as follows:

- The number of subsets *M* into which the complete set of pixels is partitioned is set to 40. Concerning *M*, it is evident that the final sub-images (needed for the analysis) cannot be too small, otherwise they do not contain enough of information. On the other hand, they should not be unnecessarily too large otherwise the procedure becomes computationally too costly. Our numerical experiments show that the size of sub-images 0.3-0.4 MP is suitable for our goals, leading to $M\approx 40$. Evidently, changing the value of *M* can influence the results but it should be set (tuned) carefully. On the other hand, if once reasonably set for a class of specific images, it appears that it is not necessary to change it from one image to another.
- The number of possible sample sizes is set to 28. Thus, the different sizes range from 100 to $318\,187\ (=N/M)$ pixels. The set of sample sizes $\mathscr {S}$ is composed of $\Big \{\bigcup _{i=1}^5 {10^2\cdot i}, \bigcup _{i=1}^5 {10^3\cdot i},\ \bigcup _{i=1}^5 {10^4\cdot i},\ \bigcup _{i=1}^6 {\big (4\cdot 10^4\cdot i + 6\cdot 10^4\big )},\ \bigcup _{i=2}^4 {7\cdot 10^i},\ \bigcup _{i=1}^3 {15\cdot 10^i},\ 318\,187\Big \}$.
- For each sample size $r_{ms_{j}^{*}}$, $m \in (1,\ldots ,M)$, the function $\mathscr {C}(\cdot )$ is estimated $B=100$ times.
- The ratio $\pi$ between the cardinalities of the learning set and validation set is set to 4.
- *Classification And Regression Trees* (CART^39^) are used as the reference classifier $\kappa$ in the validation experiment Note that, in principle, any binary classifier might be used within PARSEG. We use CART as it is flexible, capable of dealing with collinearity effects, detecting complex interaction effects, and processing high dimensional data sets. At the same time, it rarely induces overfitting problems and it is well known for its good predictive capabilities.

**Figure 2:**
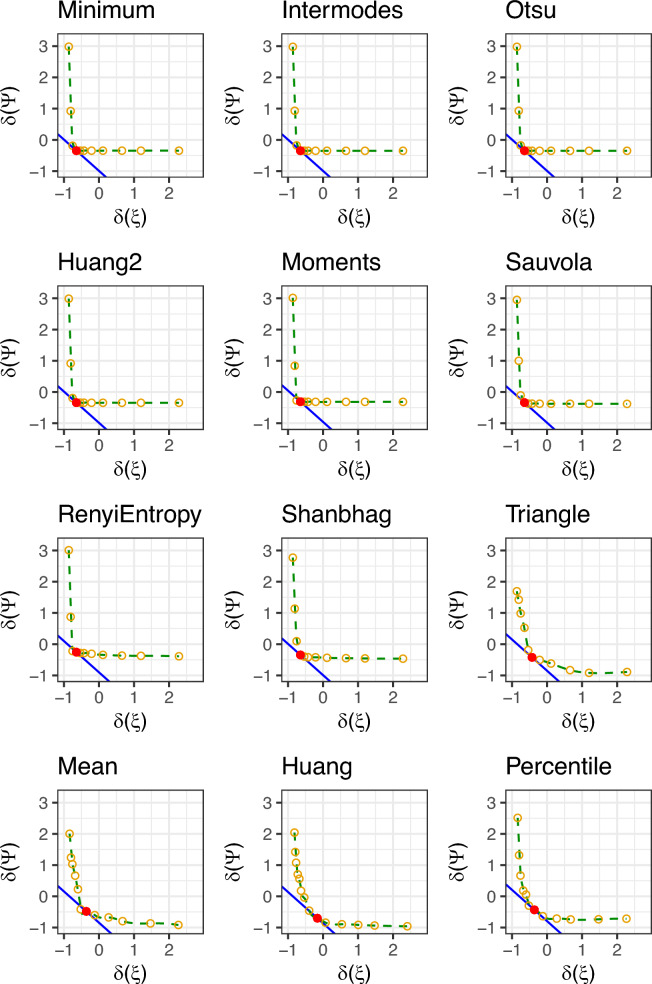
For each segmentation algorithm, the projection of the points of $\mathscr {D}_j$ identified by the standardized sample sizes $\delta (\xi )$ (*x*-axis), where $\xi$ is the subset of sample sizes needed to find the optimal sample size $s^*$, and the standardized consistency measures $\delta (\Psi )$ (*y*-axis). The dashed line represents the cubic spline that estimates their relationship. The solid line identifies the tangent of the cubic spline, i.e, the point where its derivative equals $-1$, while the red point has coordinates $(s^*_j,\widehat{\psi }_{s^*_j})$: it corresponds to the point closest to the tangent line.

The output of the procedure described in Section “﻿PARSEG﻿” aimed at determining the optimal sample size for the image validation experiment is shown in Fig. 2. For each segmentation algorithm, the optimal size is selected according to Eq. (2), and the quality of the validation experiment is measured by computing the average sensitivity metric introduced in Eq. (7). Table 3 provides evidence about the reduction of the execution times induced by the proposed method. The total number of pixels used in the analysis (*sampling* size) ranges from 2.67% to 3.16% of the total number of pixels composing the entire image, the value depending on the segmentation algorithm. The proposed approach allows us to save from 85% to 93% of the time required to perform statistical validation on the entire segmented image. The time saved is indicated by $\Delta$ and computed as follows

8 $$\begin{aligned} \Delta = \frac{(\mathscr {T}_i - \mathscr {T}_s)}{\mathscr {T}_i }\cdot 100\% \end{aligned}$$

where $\mathscr {T}_i$ is the time required to validate the results of the binary segmentation carried out on the entire image and $\mathscr {T}_s$ is the time required to validate the results of the binary segmentation through PARSEG. The difference in the computational time among segmentation algorithms in our case is due solely to the time needed to estimate the optimal sample size. In particular, the time for estimating the optimal sample size depends on how close the segmentation output obtained by the segmentation algorithm is to the pattern expressed by the color channels. More precisely, if the segmentation output differs substantially from the pattern expressed by the color channels (i.e., the original image), PARSEG needs more time to reach its stopping criterion in the optimal sample size estimation.

**Table 3:** Sizes used to perform the proposed approach for each segmentation algorithm and the corresponding computational time obtained for the *Giallo Bosa* image.

Segmentation algorithm	Sampling size	Sampling size as % of entire image	Computational time		
			Sample (opt. size + $M-1$ samples)	Whole	$\Delta$
Minimum	339 737	2.67%	32 (9 + 23)	217	85%
Intermodes	339 737	2.67%	32 (9 + 23)	218	85%
Otsu	339 737	2.67%	32 (9 + 23)	218	85%
Huang2	339 737	2.67%	32 (9 + 23)	217	85%
Moments	339 737	2.67%	33 (10 + 23)	216	85%
Sauvola	339 737	2.67%	33 (10 + 23)	237	86%
RenyiEntropy	339 737	2.67%	38 (15 + 23)	493	92%
Shanbhag	339 737	2.67%	36 (13 + 23)	373	90%
Triangle	347 537	2.73%	38 (15 + 23)	489	92%
Mean	359 337	2.82%	39 (16 + 23)	486	92%
Huang	402 537	3.16%	41 (18 + 23)	462	91%
Percentile	359 337	2.82%	41 (18 + 23)	578	93%

The second and third columns report the numbers of pixels used and the percentages of pixels of the complete image, respectively. The last three columns show the times (in minutes) needed to carry the analyses out using the proposed approach (sample), on the entire image (whole) and the savings using the proposed approach ($\Delta$). Concerning the proposed approach in brackets the decomposition of the time into its two components: the time needed to selected the optimal sample size (opt. size) and that to carry out the analysis in the remaining $M-1$ samples.

To demonstrate the effectiveness of PARSEG, its performance is compared to that obtained without applying it. To carry out this comparison, the segmentation outcomes of all twelve binary segmentation algorithms are validated using the total number of pixels *N*. The main results are summarized in Table 4. For both approaches to the validation, the global average sensitivity of the segmentation outputs stemming from the use of different algorithms is sorted in decreasing order. Note that the average sensitivity substantially preserves the same ranking of the segmentation outputs if validation is performed on the entire image or the optimal size is used. Next, the similarity between the two rankings is measured with the rank correlation coefficient $\tau _X$^40^, an extended version of Kendall’s $\tau$^41^, where ‘X’ stands for extended. The coefficient $\tau _X$ takes on values in $[-1,+1]$: $\tau _X=+1$ if the two rankings are identical; $\tau _X=-1$ if they are perfectly opposed. If no correlation exists between the two rankings, then $\tau _X=0$. In our case, $\tau _X =0.939$ confirms the high similarity between the two rankings. The performance of the two approaches is further described in relative terms (the columns *Normalized* $\bar{\phi }$ in Table 4) to simplify their comparison. It is evident that the two approaches can be considered equivalent with respect to the overall quality of the validation experiment. The use of a Spearman correlation coefficient gives very similar results.

**Table 4:** *Giallo Bosa* image: Comparison of the validation of all twelve segmentation outcomes performed on the optimal sample selected by the proposed approach (sample) or on the entire image (whole).

Segmentation	$\bar{\phi }$ (Rank)		Normalized $\bar{\phi }$	
algorithm	Whole	Sample	Whole	Sample
Minimum	0.999 95 (1)	0.999 93 (1)	1.000 00	1.000 00
Intermodes	0.999 94 (3)	0.999 90 (2)	0.999 71	0.998 96
Otsu	0.999 94 (2)	0.999 90 (3)	0.999 72	0.998 87
Huang2	0.999 57 (4)	0.999 26 (4)	0.982 35	0.978 60
Moments	0.999 53 (5)	0.999 19 (5)	0.980 82	0.976 53
Sauvola	0.998 19 (6)	0.998 16 (6)	0.918 44	0.943 62
RenyiEntropy	0.998 15 (7)	0.996 24 (7)	0.916 91	0.882 83
Shanbhag	0.997 69 (8)	0.995 12 (8)	0.895 40	0.847 18
Triangle	0.995 56 (10)	0.993 46 (9)	0.796 72	0.794 63
Mean	0.997 37 (9)	0.991 77 (10)	0.880 77	0.741 07
Huang	0.991 74 (11)	0.984 06 (11)	0.620 15	0.496 40
Percentile	0.978 34 (12)	0.968 42 (12)	0.000 00	0.000 00

The average sensitivities and their ranks (in parenthesis) are reported together with their normalized values obtained by rescaling average sensitivities to [0, 1].

**Figure 3:**
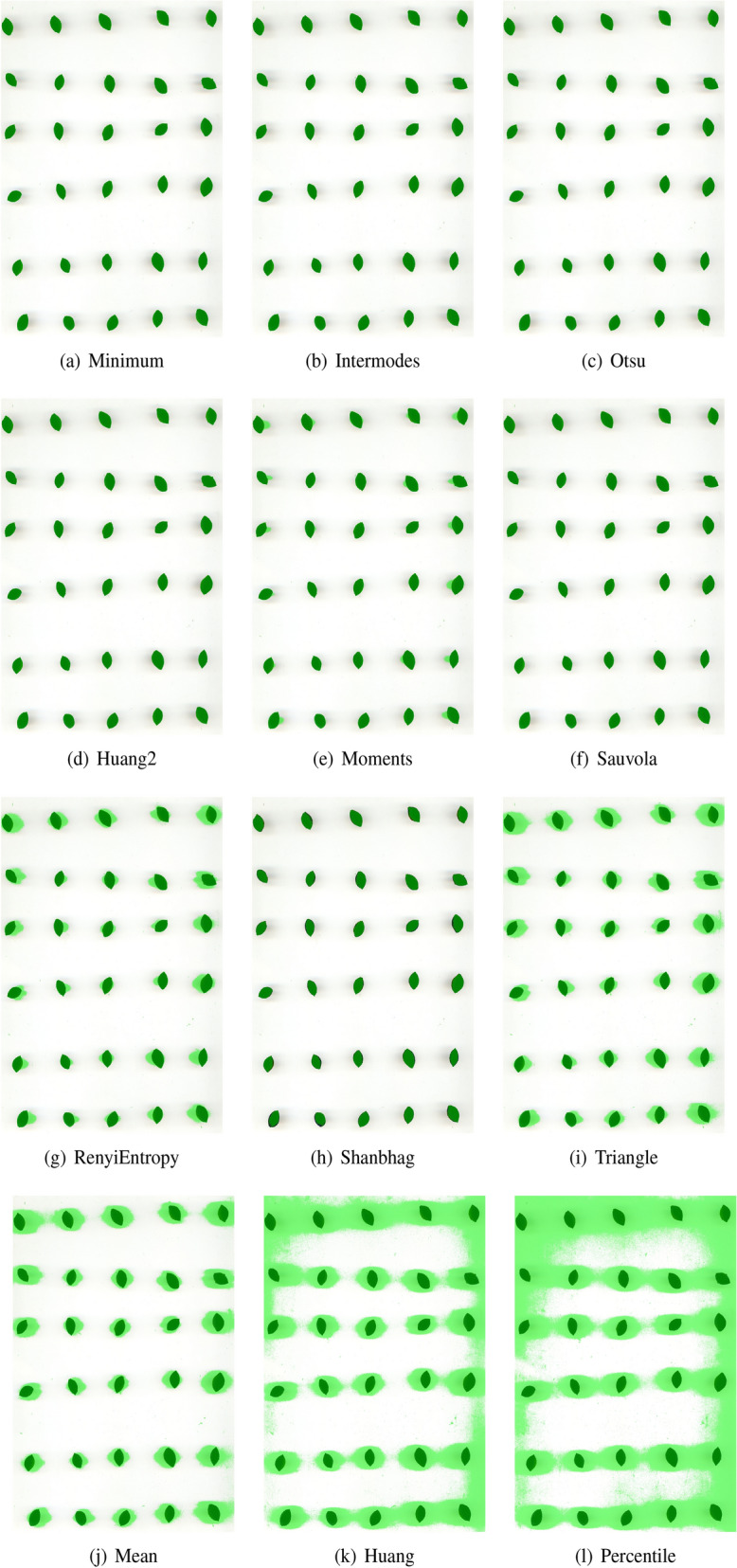
Output of considered segmentation methods obtained for the *Giallo Bosa* image. Pixels plotted in green correspond to those recognized as foreground by the given segmentation algorithm.

For the sake of completeness, Fig. 3 shows the output obtained from the binary segmentation methods used. The green points correspond to the pixels that have been recognized as the foreground by the specific segmentation algorithm. The images are ordered according to the quality (sensitivity) of the validation experiment. It is worth noticing that, consistent with the results reported in Table 4, the first four segmentation settings provide valuable outputs if compared with the other ones.

**Figure 4:**
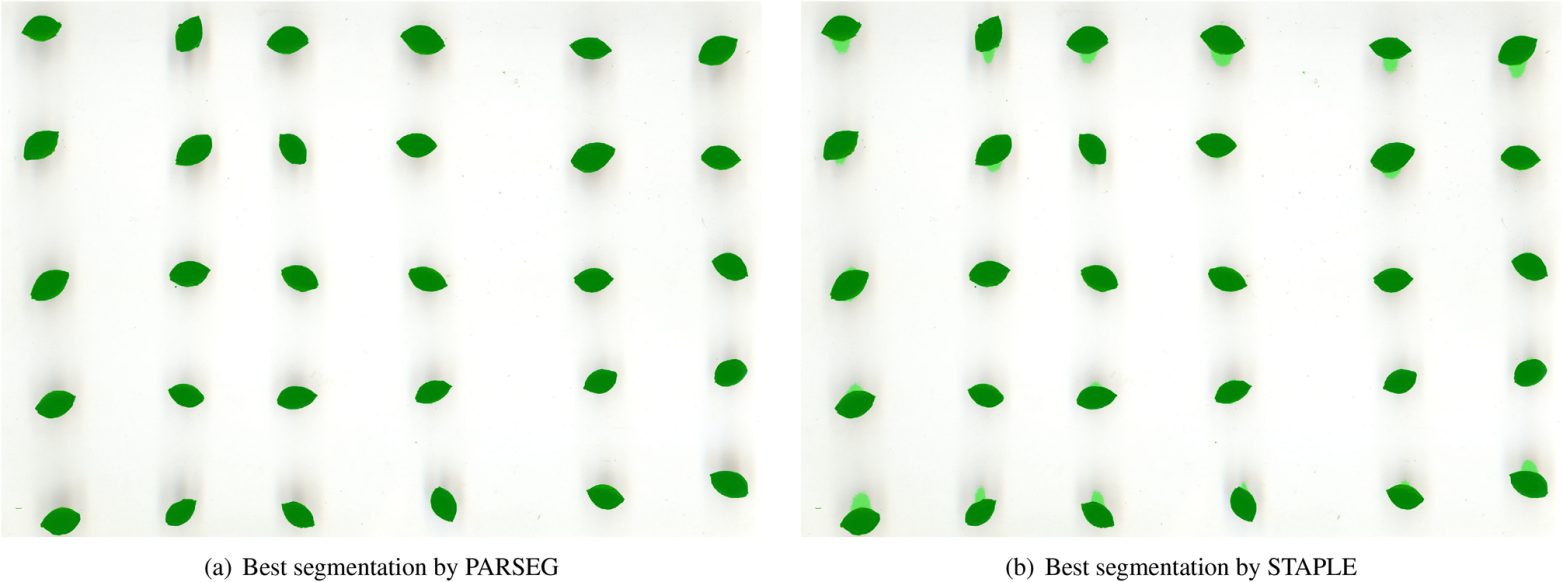
Best segmentation obtained by PARSEG and STAPLE methods for the *Giallo Bosa* image. Pixels plotted in green correspond to those recognized as foreground by the given segmentation algorithm.

Finally, the performances of PARSEG and STAPLE are compared in Fig. 4, where the best segmentation obtained by the segmentation algorithms for the former and the segmentation output estimated by the latter are shown. Since the true segmentation is unknown, it is impossible to assess which the best method is with no uncertainty, but it appears that the result obtained by PARSEG is clearly better than that obtained by STAPLE.

We think PARSEG could not work properly in two cases. Firstly, the idea of the statistical validation of image segmentation algorithms behind PARSEG concerns the capability of the statistical classifier to recognize the pattern of separation between background and foreground inside the original image. Consequently, the choice of the statistical classifier is very important and crucial for obtaining satisfying results. Secondly, the operation of PARSEG is regulated by the partition of the data into *M* subsets characterized by a similar distribution of the categories of $\textbf{y}$ and an unknown function that maps $\textbf{x}$ to $\textbf{y}$. If the number of pixels is high (as in most cases) we expect with a high level of confidence that stratified random sampling will enforce this condition. If the number of pixels was low, however, the degree of confidence could drop. It is important to note that the former is handled by the researcher, whilst the latter is not.

### Results for different types of seeds

The same experiment presented in Section “*Giallo Bosa* example” is repeated for the other 15 images of different seed species. Table 5 reports the results obtained on all 16 images. It has turned out that the average of the sampling size considering the segmentation algorithms, i.e., the entire set of pixels, ranges from 314 737 to 474 332, reducing the computational complexity induced by PARSEG on average below 4% of the total number of pixels composing the entire image. Specifically, PARSEG allows us to save from 86% to 92% of the time compared to the time required when performing validation using all pixels. The appropriateness of PARSEG is further confirmed by the high values of the $\tau _X$ coefficient, which range from 0.818 to 0.970.

**Table 5:** Results obtained for the images of all sixteen of the analyzed seed species.

Seed species	*N*	Average sample size	Average % whole image	Average comput. time			$\tau _X$
				Sample image	Whole image	$\Delta$	
*Cariadoggia*	12 477 201	347 922	2.79%	36.25	435.0	92%	0.879
*Cariasina*	11 569 761	329 669	2.85%	33.17	318.5	90%	0.970
*Coru*	12 491 721	385 793	3.09%	36.33	336.8	89%	0.970
*Coru e Columbu*	12 090 111	418 852	3.46%	43.42	344.8	87%	0.909
*Croccorighedda*	12 077 091	347 902	2.88%	34.67	377.4	91%	0.939
*Fara*	12 821 281	377 349	2.94%	37.50	362.0	90%	0.939
*Giallo Bosa*	12 727 494	348 887	2.74%	35.58	350.3	90%	0.939
*Laconi A*	12 898 821	398 287	3.09%	38.42	379.3	90%	0.939
*Melone*	12 738 291	474 332	3.72%	44.83	329.4	86%	0.879
*Mirabolano Giallo*	11 206 801	314 737	2.81%	32.17	331.5	90%	0.879
*Mirabolano Rosso*	12 374 131	353 261	2.85%	35.08	313.8	89%	0.879
*Nero Sardo*	13 072 930	374 498	2.86%	37.58	355.9	89%	0.879
*San Giovanni*	10 943 511	350 362	3.20%	31.17	243.0	87%	0.818
*Sanguigna I Bosa*	12 233 641	360 474	2.95%	35.33	351.5	90%	0.970
*Shiro*	13 273 416	431 660	3.25%	39.67	356.2	89%	0.879
*Sighera*	12 333 561	369 697	3.00%	36.08	370.1	90%	0.909

The first column reports the seed species, the second column the numbers of pixels that compose the images, and the third and forth columns indicate the average values of, respectively, the sampling size and the percentage of pixels used from the entire image considering the twelve segmentation algorithms. The fifth to the seventh columns show the average times (in minutes) needed to carry out the analyses using the proposed approach (sample), the entire image (whole) and the percentage decrease in computing time obtained when using the proposed approach ($\Delta$). The last column reports the $\tau _X$ coefficients computed considering the rankings obtained using the proposed approach and those using all pixels.

**Figure 5:**
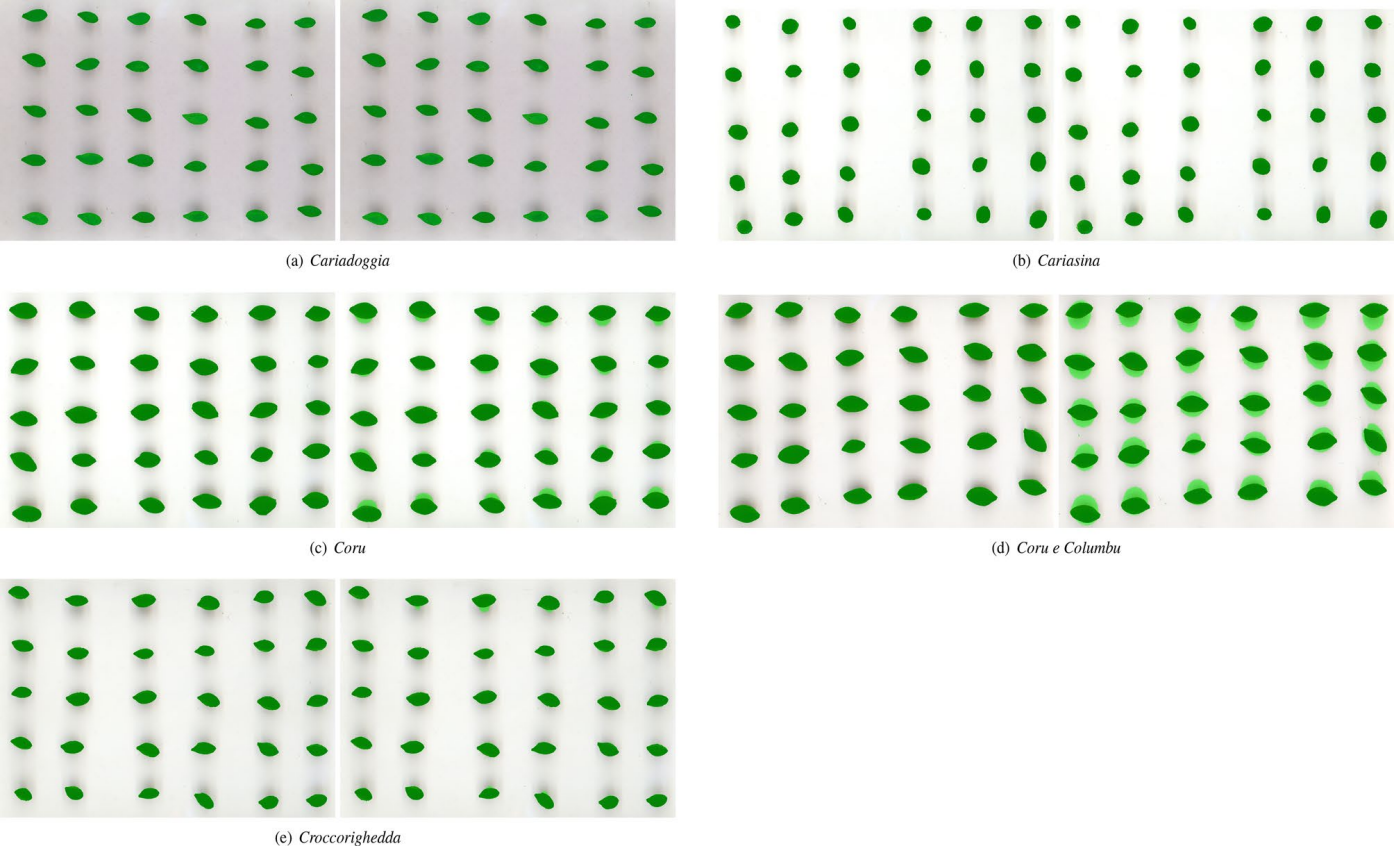
Best segmentations obtained by PARSEG (on the left) and STAPLE (on the right) for the images: *Cariadoggia*, *Cariasina*, *Coru*, *Coru e Columbu*, *Croccorighedda*. Pixels plotted in green correspond to those recognized as foreground by the given segmentation algorithm.

**Figure 6:**
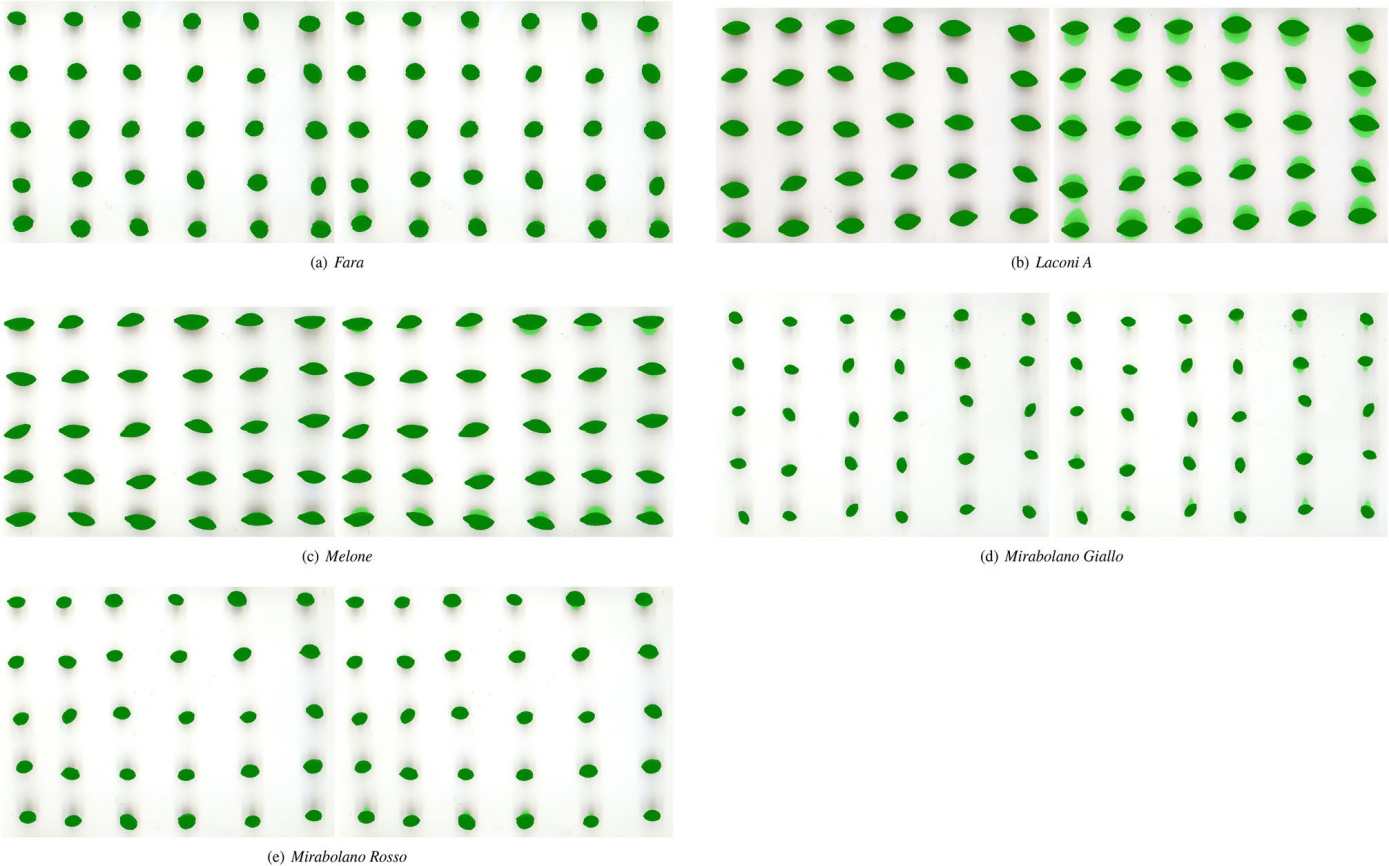
Best segmentations obtained by PARSEG (on the left) and STAPLE (on the right) for the images: *Fara*, *Laconi A*, *Melone*, *Mirabolano Giallo*, *Mirabolano Rosso*. Pixels plotted in green correspond to those recognized as foreground by the given segmentation algorithm.

**Figure 7:**
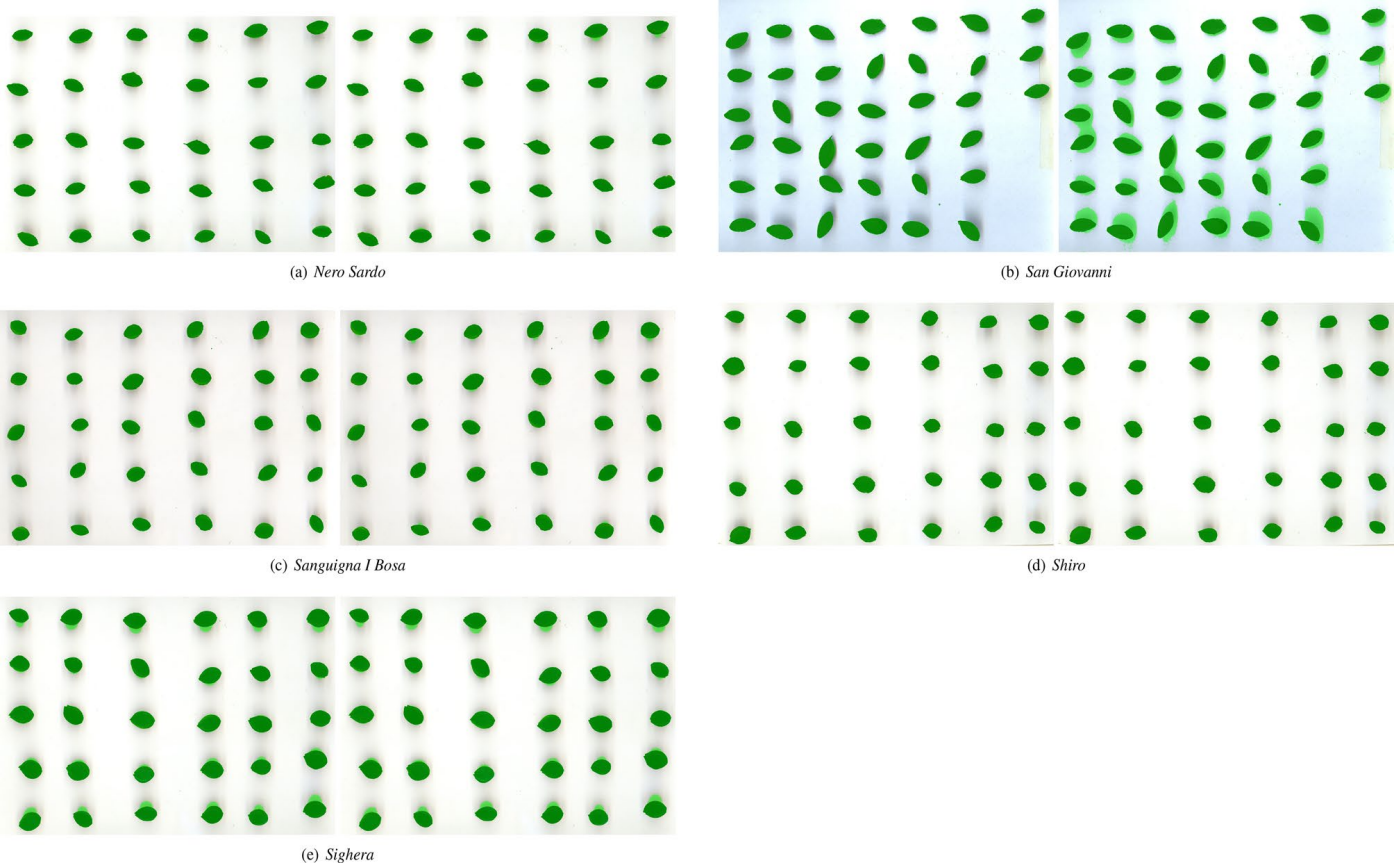
Best segmentations obtained by PARSEG (on the left) and STAPLE (on the right) for the images: *Nero Sardo*, *San Giovanni*, *Sanguigna I Bosa*, *Shiro*, *Sighera*. Pixels plotted in green correspond to those recognized as foreground by the given segmentation algorithm.

Figures 5, 6 and 7 compare the best segmentations obtained by PARSEG and STAPLE for the 15 additional images. PARSEG obtained a better segmentation 11 times over 15 (73%), whilst no important differences in results are observed the remaining four times.

## Concluding remarks

To reduce the computational complexity of statistical validation for binary segmented images, PARSEG has been introduced as a novel statistical technique. The suggested approach preserves the performance of the system validation experiment and considerably reduces computational complexity. Its main features are the use of a classifier and related performance metric enabling one to validate the output of binary segmentation algorithms. Although sensitivity has been used as the metric for classifier performance as a viable default choice, it is possible to use different metrics as well. Ability to perform statistical validation on a reduced sample of pixels while providing the same results as when the validation is carried out using all available pixels, the use of smoothing splines to select the reduced optimal sample and the consistent reduction of the computational complexity belong among its main advantages.

We applied PARSEG in a relatively simple framework (the segmentation of seed images). When validating images composed of about 13 million pixels in total, PARSEG used a sample size below 4% of the full image size (on average) to obtain validation results that were fully comparable to those obtained when all pixels were used for validation. As a result, the computing time required to perform image validation using all pixels was reduced by approximately 90%. The advantages of using PARSEG are greater when analyzing images of the same type.

## Future work

In this paper we concentrate especially on binary images. In the future, we plan to study in detail two points. The first one is how the suggested approach behaves when segmentation algorithms partition the image into multiple parts and not in a binary way, and what and how should be appropriately modified. The second is to study in detail the influence of different metrics when PARSEG is applied to different types of images.

## Figures and Tables

**Figure 8:**
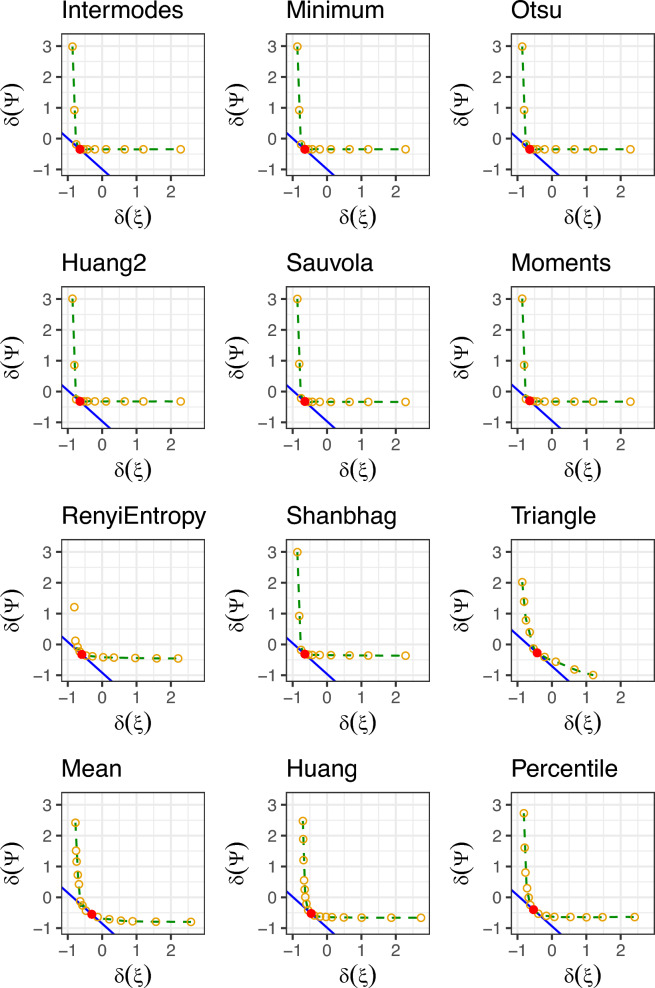
For each segmentation algorithm, the projection of the points of $\mathscr {D}_j$ identified by the standardized sample sizes $\delta (\xi )$ (*x*-axis), where $\xi$ is the subset of sample sizes needed to find the optimal sample size $s^*$, and the standardized consistency measures $\delta (\Psi )$ (*y*-axis). The dashed line represents the cubic spline that estimates their relationship. The solid line identifies the tangent of the cubic spline, i.e, the point where its derivative equals $-1$, while the red point has coordinates $(s^*_j,\widehat{\psi }_{s^*_j})$: it corresponds to the point closest to the tangent line.

## Data Availability

Data and source code of the analysis in R are available from the authors on request by contacting the corresponding author at frigau@unica.it

## Appendix: Alternative metrics: F1 score

In the appendix we provide the results obtained for *Giallo Bosa* if other metrics were used as the reference classifier performance. In particular, we consider F1 score metric. Table 6 summarizes the results of the comparison of the validation of all twelve segmentation outcomes performed on the optimal sample selected by the proposed approach (sample) or on the entire image (whole). Instead, Fig. 8 shows the output of the procedure described in Section “﻿PARSEG﻿” aimed at determining the optimal sample size for the image validation experiment.

**Table 6:** *Giallo Bosa* image: Comparison of the validation of all twelve segmentation outcomes performed on the optimal sample selected by the proposed approach (sample) or on the entire image (whole). The average F1 score and their ranks (in parenthesis) are reported together with their normalized values obtained by rescaling average F1 score to [0, 1].

Segmentation algorithm	$\bar{\phi }$ (Rank)		Normalized $\bar{\phi }$	
	Whole	Sample	Whole	Sample
Intermodes	0.999 95 (2)	0.999 88 (1)	0.999 94	1.000 00
Minimum	0.999 96 (1)	0.999 87 (2)	1.000 00	0.999 63
Otsu	0.999 95 (3)	0.999 87 (3)	0.999 89	0.999 61
Huang2	0.999 73 (4)	0.999 52 (4)	0.989 76	0.988 66
Sauvola	0.999 06 (6)	0.998 87 (5)	0.959 90	0.968 42
Moments	0.999 47 (5)	0.998 39 (6)	0.978 24	0.953 49
RenyiEntropy	0.997 45 (8)	0.996 15 (7)	0.887 40	0.884 01
Shanbhag	0.998 42 (7)	0.995 95 (8)	0.930 90	0.877 73
Triangle	0.996 57 (9)	0.993 34 (9)	0.847 85	0.796 58
Mean	0.996 36 (10)	0.992 40 (10)	0.838 18	0.767 36
Huang	0.988 31 (11)	0.983 23 (11)	0.476 12	0.482 41
Percentile	0.977 72 (12)	0.967 71 (12)	0.000 00	0.000 00
